# Effect of Carbon Black Content and Firing Atmosphere on the Properties and Microstructure of Al_2_O_3_-SiC-C Castables

**DOI:** 10.3390/ma17225506

**Published:** 2024-11-12

**Authors:** Quanli Jia, Jing Chen, Mantang He, Mengyang Sang, Pingyi Zhou, Haoxuan Ma

**Affiliations:** School of Materials Science and Engineering, Henan Key Laboratory of High Temperature Functional Ceramics, Zhengzhou University, Zhengzhou 450001, China; jiaquanli@zzu.edu.cn (Q.J.); chenjing202312@163.com (J.C.); hemantang111@gmail.com (M.H.); sangmengyang@gs.zzu.edu.cn (M.S.); zhou_py@aactechnolojies.com.cn (P.Z.)

**Keywords:** Al_2_O_3_-SiC-C castables, carbon black, firing atmosphere, melting furnace, fly ash

## Abstract

Al_2_O_3_-SiC-C (ASC) castables containing spherical asphalt are widely utilized in high-temperature metallurgical furnaces because of their good abrasive resistance and slag resistance; however, the release of hazardous benzopyrene during the pyrosis process in spherical asphalt is detrimental to the environment and to the health of furnace workers. Herein, nontoxic nano carbon black (CB) was selected as the carbon source for ASC castables, and the effects of the CB amount and sintering atmosphere on the properties of ASC castables were investigated in this work. The results show that on increasing CB from 0.5% to 2%, the cold strength of the samples after firing in the reducing atmosphere increased, the residual strength increased, and the slag penetrated depth decreased; the reasons can be ascribed to nano CB being able to fill the pores to reduce the apparent porosity of the castables. Furthermore, SiC whiskers were formed at elevated temperatures and generated a network structure, which was beneficial in improving their properties. When CB was 1%, the cold modulus of the rupture of the samples after firing in the oxidizing atmosphere and reducing atmosphere were higher (about 20 MPa), the retained strength ratio of the samples pre-fired in the reducing atmosphere was the highest (85.4%), the hot strength at 1400 °C of the samples tested in the oxidized atmosphere was the highest (5.3 MPa), and the slag resistance of the samples measured in the oxidizing atmosphere was the best. The castables heat-treated in the air atmosphere possessed higher hot strength and slag resistance; the reasons can be attributed to the formed SiO_2_ derived from the oxidation of SiC, which reacted with Al_2_O_3_ to form mullite, creating a strengthening effect and decreasing the porosity and increasing the viscosity of slag, thereby improving the hot strength and slag resistance.

## 1. Introduction

In 2019, 235.6 million tons of municipal solid waste and 44.99 million tons of hazardous solid waste were generated in China [1]. The rapid and efficient treatment of this solid waste is conducted by high-temperature incineration, because a large volume reduction (~90%), chemical stability, and low cost can be achieved [2,3]. Unfortunately, approximately 10 million tons of bottom ashes and fly ashes are produced during the incineration process for municipal solid waste [4]. Compared to bottom ashes, fly ashes contain leachable heavy metals, soluble salts, chloride, and noxious organic dioxin, etc., and are detrimental to human health and the environment; therefore, they are categorized as solid hazardous waste [5]. Prior to landfilling, these fly ashes need further treatment by solidification/stabilization [6]. It is difficult to safely landfill these huge amounts of ash residues because of limited landfill resources; therefore, finding an effective treatment for fly ashes represents an urgent problem to be solved [7,8]. To respond to this issue, thermal melting techniques [9,10], including electric arc furnace, DC arc plasma, plasma furnace, and steel-making electric arc furnace, have been used to safely and effectively handle these fly ashes because they offer several advantages, such as volume reduction, detoxification, and resource recycling [11]. However, refractory linings for slag melting furnaces have repeatedly suffered from severe damage, including complicated erosion by slag, slats, and gaseous products, thermal shock, and wear stress, resulting in a lower service life in refractories and thereby greatly restricting the utilization of these melting techniques [12]. To date, Al_2_O_3_-Cr_2_O_3_-based refractories have been utilized in waste melting furnaces because they have higher abrasive resistance and excellent corrosion resistance; however, they suffer from severe thermal spalling derived from larger temperature gradients and the mismatch between slag penetration layers and refractories. Furthermore, toxic Cr^6+^ can be formed in their service condition, which may endanger the environment [13]. These factors inhibit the application of Cr_2_O_3_-containing refractories; therefore, high-performance Cr_2_O_3_-free refractories are the best choice for slag melting furnaces [13,14].

Some works have found that SiC-bearing castables have many merits, including better thermal shock resistance, good salt corrosion resistance, and higher strength, and can be applied in melting furnaces [15,16]. To further improve their high-temperature properties, carbonaceous materials, including spherical asphalt [17] and carbide-coated graphite, have been utilized in SiC-bearing castables [18,19,20,21,22]. As an example, spherical asphalt has been commonly utilized in castables because of its low cost and good hydrophilic and dispersive properties. Unfortunately, the release of benzo (a) pyrene during its pyrolysis process is harmful to the environment, and it should be replaced by a nontoxic carbon source [17]. Coated graphite offers much better dispersibility and oxidation resistance; however, the higher water demand, higher apparent porosity, and high costs of the castables containing coated graphite represent issues that noticeably limit its application [18,19].

To enhance the properties of carbon-containing castables, researchers have attempted to utilize a number of carbon materials in the castables. For instance, the thermal shock resistance (TSR) and slag resistance of Al_2_O_3_-SiC-C (ASC) castables were improved by adding modified coal tar pitch in our previous work [23]. Li et al. [24] found that the flowability and mechanical properties of ASC castables were improved using carbon microspheres fabricated by the hydrothermal carbonization method instead of spherical asphalt. Zang et al. [25] demonstrated that the oxidation resistance and TSR of ASC castables were significantly improved by adding carbon nanotube/calcium aluminate cement composite powders. However, these carbonaceous materials have some drawbacks, including the release of harmful compounds, a high cost, and low production levels, which have also greatly restricted their utilization. Carbon black (CB) is characterized by a low specific surface area, single spherical particles, high purity (>99%), a small particle size (<0.5 μm), and a low surface oxygen content (<0.3%) and can be applied in carbon-containing castables because of its easy dispersion and good fluidity [26]. Wu et al. [27] found that the corrosion resistance of ASC castables could be enhanced by adding CB, but the addition of CB reduced their physical properties.

More importantly, the castable linings were exposed to different practical working areas, including the upper surface, in the ambient environment atmosphere, i.e., the oxidizing atmosphere (OA); the intermediate area in contact with the solid–liquid interfaces of molten slag, i.e., the weak-oxidizing atmosphere (WOA); and the surface below the air–liquid interface, i.e., the reducing atmosphere (RA) [23,28]. The service life and high-temperature properties of ASC castables are also influenced by their service conditions [23,28], and herein, we also studied their properties after heat treatment in different atmospheres. Aiming to enhance the properties of ASC castables, many works have been conducted via adding carbon fibers [29], Al-Si alloy powders [30], aluminum fibers [31], ammonium metatungstate [32], etc. In this work, nano CB was introduced to ASC castables, and the influence of CB amounts on the crystal phase and microstructural characterization of the castables after firing in various atmospheres was investigated. The results demonstrated that the physical properties, hot strength, TSR, and slag resistance of the ASC castables were noticeably influenced by the CB content and firing atmospheres, and their improvement mechanisms were also illustrated.

## 2. Experimental Procedures

Material precursors including fused brown alumina aggregates (Al_2_O_3_ > 95%, 5–3 mm, 3–1 mm), SiC aggregates and fines (SiC > 97.5%, 1–0 mm and ≤0.074 mm), ultrafine α-Al_2_O_3_ (Al_2_O_3_: 99.7%, d50 = 1.2 μm), microsilica (SiO_2_ > 95%, 951U), Si fines (Si > 98%, ≤0.074 mm), calcium aluminate cement (Secar 71), and carbon black (N990, C > 99%) were used in this study.

Raw materials were fabricated according to Table 1, and then the castables were produced via mixing with 4.2% water and casted in 25 mm × 25 mm × 150 mm bar samples and crucible samples (Φ70 mm × 70 mm with a hole Φ35 mm × 40 mm). They were cured at room temperature and dried at 110 °C for 24 h; afterwards, the bar samples were heat-treated for 3 h at 1100 °C, 1300 °C, and 1500 °C in the OA, WOA, and RA, respectively [23,28].

Physical properties including the cold modulus of rupture (CMOR) and permanent linear change (PLC), apparent porosity (AP), and bulk density (BD) of the castables were measured according to China’s standards. The TSR of the samples pre-fired at 1500 °C in different atmospheres were determined (ΔT = 1100 °C, three air-cooled cycles), the residual CMOR (CMORst) was tested, and the residual strength ratio (RSR) was evaluated according to this Equation: RSR = CMORst/CMOR × 100%. The hot strength (HMOR) of the sample after drying at 110 °C was measured at 1400 °C for 0.5 h in different firing atmospheres (RA, OA, and WOA). The slag resistance of the castables was assessed using 12 g fly ashes with a static crucible at 1500 °C for 3 h in different treatment atmospheres; the corrosion index was calculated via this Equation: I_c_ = (A_c_/A_0_) × 100%, where I_c_ was the corrosion index, A_c_ was the corrosion area, and A_0_ was the crucible hole area, which were calculated by IPP6.0 software (Media Cybernetics, Baltimore, MD, USA) and presented in our previous works [23,28].

The pore size distribution of the samples was characterized using Mercury Porosimetry (Autopore IV9500, Micromeritics Instrument Co, Atlanta, GA, USA). Phase characteristics analysis was carried out using X-ray diffraction (XRD, Philips X’ pert, Almelo, Netherlands). The microstructure and element composition of the pre-fired samples were characterized using a Zeiss scanning electron microscope equipped with an energy-dispersive spectrometer (SEM-EDS, Zeiss EVO HD15, Oberkochen, Germany).

## 3. Results and Discussion

### 3.1. Physical Properties of the Castables

**Figure 1:**
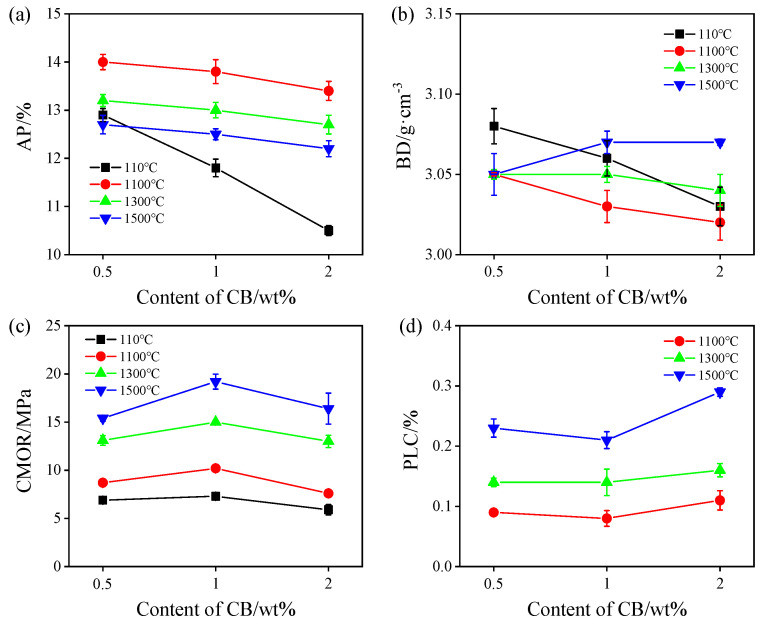
Physical properties of the samples after firing in reduced atmosphere. (**a**) Apparent porosity, (**b**) bulk density, (**c**) cold modulus of rupture, and (**d**) permanent linear change.

Figure 1 shows the physical properties of ASC castables heat-treated in the RA. As seen in Figure 1a,b, the AP values of the samples decrease with an increasing CB level from 0.5% to 2%, and the BD values of the samples heat-treated at 110 °C, 1100 °C, and 1300 °C also decrease, but they slightly increase after firing at 1500 °C, which may be attributed to nanosized CB being able to fill in the pores to reduce the porosity, whereas BD is also reduced because the density of CB is lower than that of SiC fines. The CMOR values of the samples fired at different temperatures increase with a rising CB amount from 0.5% to 1% and decrease with 2% CB addition (Figure 1c). The PLC values of the samples fired at 1100 °C~1500 °C are slightly increased and all positive, indicating that the ASC castables have a slight volume expansion, which may be owing to the castables containing large amounts of SiC. With the rise in treatment temperature, the CMOR values of the sample also increase, which may be attributed to the significant sintering effect and the formation of in situ SiC whiskers, resulting in a strengthening effect, which will be discussed in a later section.

**Figure 2:**
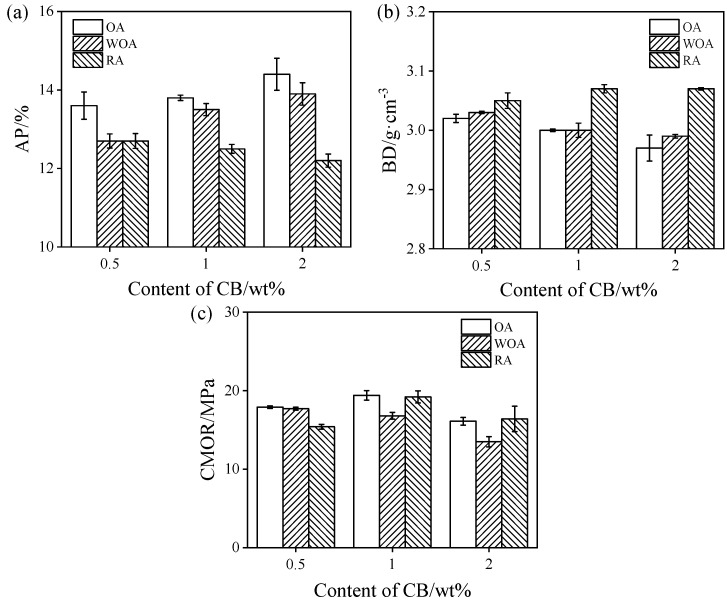
Physical properties of the samples after heat treatment in various atmospheres. (**a**) Apparent porosity, (**b**) bulk density, and (**c**) cold modulus of rupture.

The AP, BD, and CMOR values of the samples sintered in different firing atmosphere at 1500 °C are shown in Figure 2. As presented in Figure 2a,b, the AP values of the castables fired in the OA and WOA are increased, whereas BD values decrease. It is noted that the samples pre-heated in the OA have the highest AP and the smallest BD, however, the AP of the sample heat treatment in the RA is the lowest, and its BD is the largest. This may be due to CB being easily oxidized in the OA and pores being generated, thereby increasing the porosity. The oxidation of CB can be inhibited by excessive embedded carbon in the RA, and the formation of SiC whiskers is promoted in the RA because of the higher CO partial pressure; in addition, SiC whiskers are preferably formed in the pores, increasing their bulk density and reducing their AP. The CMOR values of the specimens fired in the OA and RA increase with an increase in CB from 0.5% to 1%, and decrease on a further rise in its amount to 2%; however, the CMOR of the specimens fired in the WOA is gradually reduced, as presented in Figure 2c. It can be found that CMOR of the samples with 2% CB after firing in the WOA is the lowest (15.4 MPa), which is similar to our previous work [28].

### 3.2. High-Temperature Properties of ASC Castables

**Figure 3:**
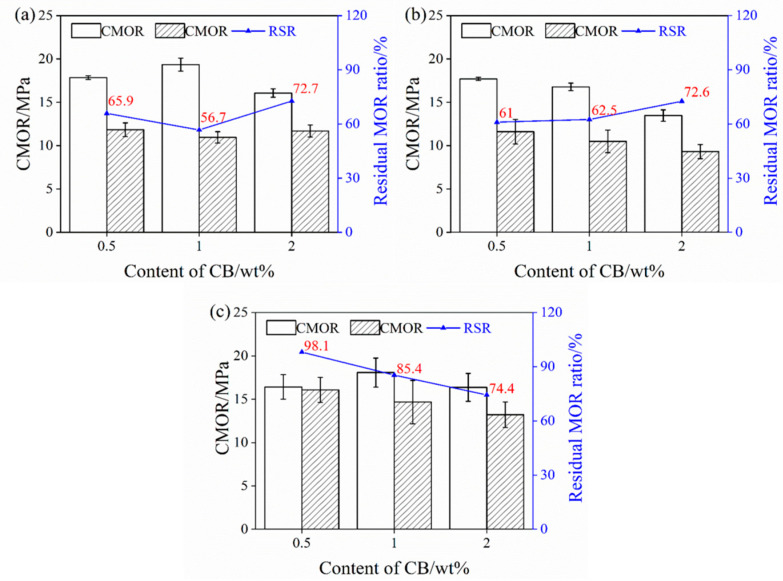
Thermal shock resistance of the samples pre-fired in different atmospheres. (**a**) OA, (**b**) WOA, and (**c**) RA.

Figure 3 shows the TSR of the samples upon increasing the CB level from 0.5% to 2%. The residual CMOR of the samples pre-fired in the OA is slightly changed (about 11 MPa), and their residual CMOR ratio decreases firstly and then increases with 2% CB, as revealed in Figure 3a. The residual CMOR of the sample pre-fired in the WOA is slightly reduced, from 10.8 MPa to 9.8 MPa, but their residual CMOR ratio increases from 61% to 72.6% (Figure 3b). As revealed in Figure 3c, the residual CMOR of the sample fired in the RA is increased; however, their residual CMOR ratio reduces from 98.1% to 74.4%. It can be found that the residual CMOR (<12 MPa) and RSR (<70%) of the sample CB1 fired in the OA and WOA are somewhat lower, while the residual CMOR (≥15 Ma) and RSR (≥85%) of the samples fired in the RA are higher. This indicates that sample CB1 fired in the RA has better TSR.

**Figure 4:**
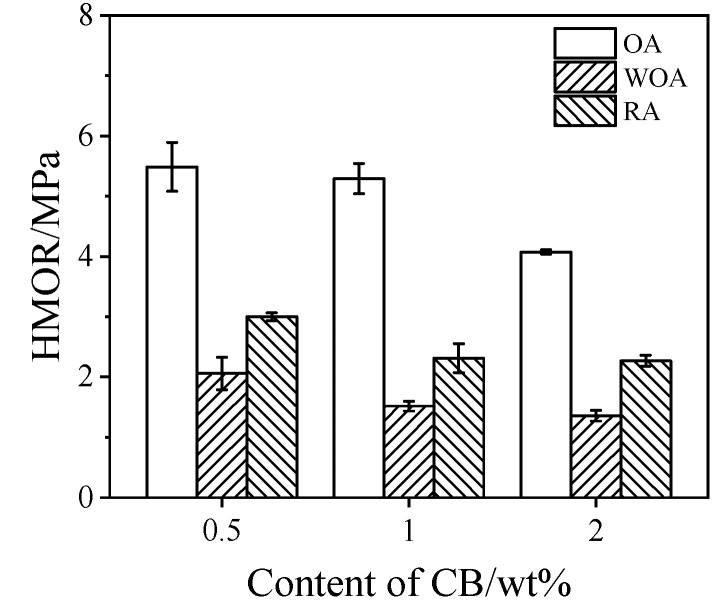
Hot modulus of rupture of ASC castables tested in various atmospheres.

The HMOR values of drying samples tested at 1400 °C in different sintering atmospheres are shown in Figure 4. On increasing the CB amount from 0.5% to 2%, the HMOR values of the samples are reduced from 5.5 MPa to 4.1 MPa when tested in the OA, from 2.1 MPa to 1.4 MPa when tested in the WOA, and from 3 MPa to 2.3 MPa when tested in the RA, respectively. The HMOR of the samples tested in the OA is the highest, and that in the WOA is the lowest, which may be related to their microstructure and phase composition.

**Figure 5:**
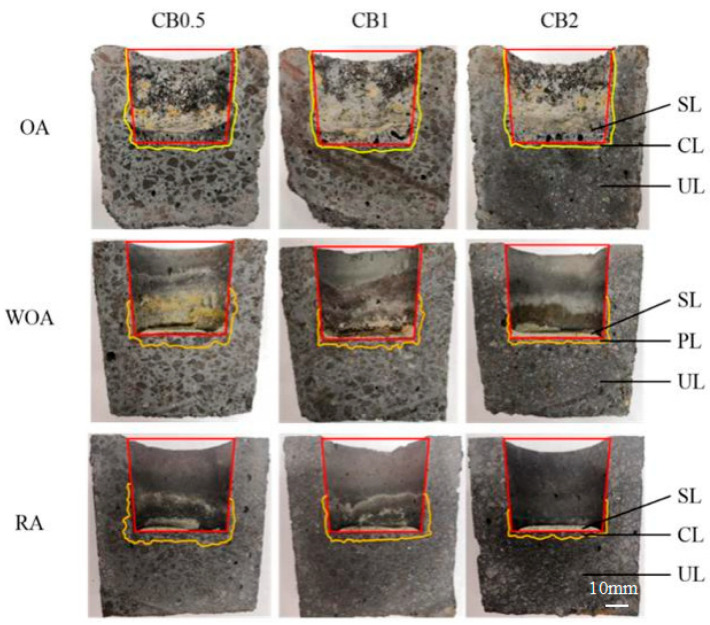
Cross-sections of the corroded samples after testing in various atmosphere (SL–slag layer; PL—slag penetrated layer; UL—uncorroded layer).

Figure 5 presents cross-sectional photos of the crucible after slag corrosion. More slag residues in the crucible tested in the OA are seen in Figure 5, and a number of pores are seen in the slags; in addition, the outline of the crucible is not clear, and obvious corrosion has occurred. A number of residual slags in the crucible later corroded in the WOA and RA, and the outline of the crucible is clear and complete, indicated that they have better slag resistance.

**Figure 6:**
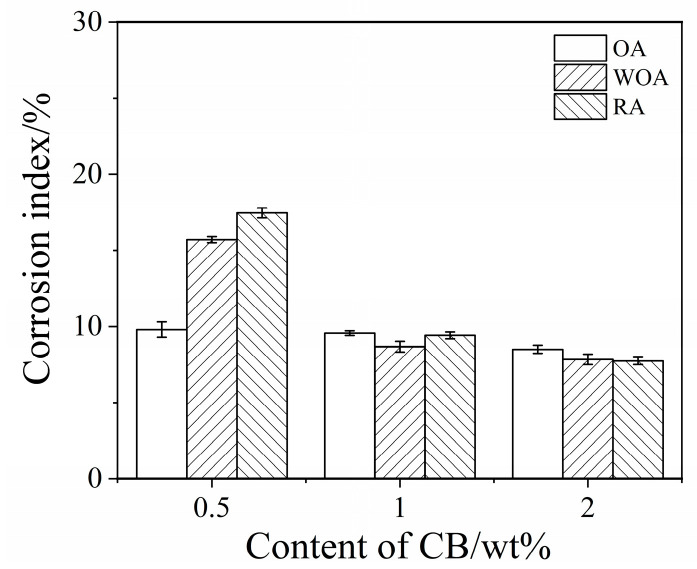
Corrosion index of ASC castables tested in various atmospheres.

The percentage corrosion index of the samples was calculated and is shown in Figure 6. On increasing the CB level, the corrosion index of the samples is slightly reduced from 10% to 8.6% in the OA, from 15.8% to 7.9% in the WOA, and from 17.5% to 7.8% in the RA, respectively. It is noted that the corrosion of the sample with 0.5% CB is the worst, especially for the sample treated in the RA. The corrosion index of sample CB2 is 8.6%, 7.9%, and 7.8%, respectively, for slag tested in the OA, WOA, and RA, indicating that the sample with 2% CB has the best corrosion resistance, which is due to more carbon addition being beneficial to slag resistance, making it better than that of the castables with adding modified coal tar pitch [23].

**Figure 7:**
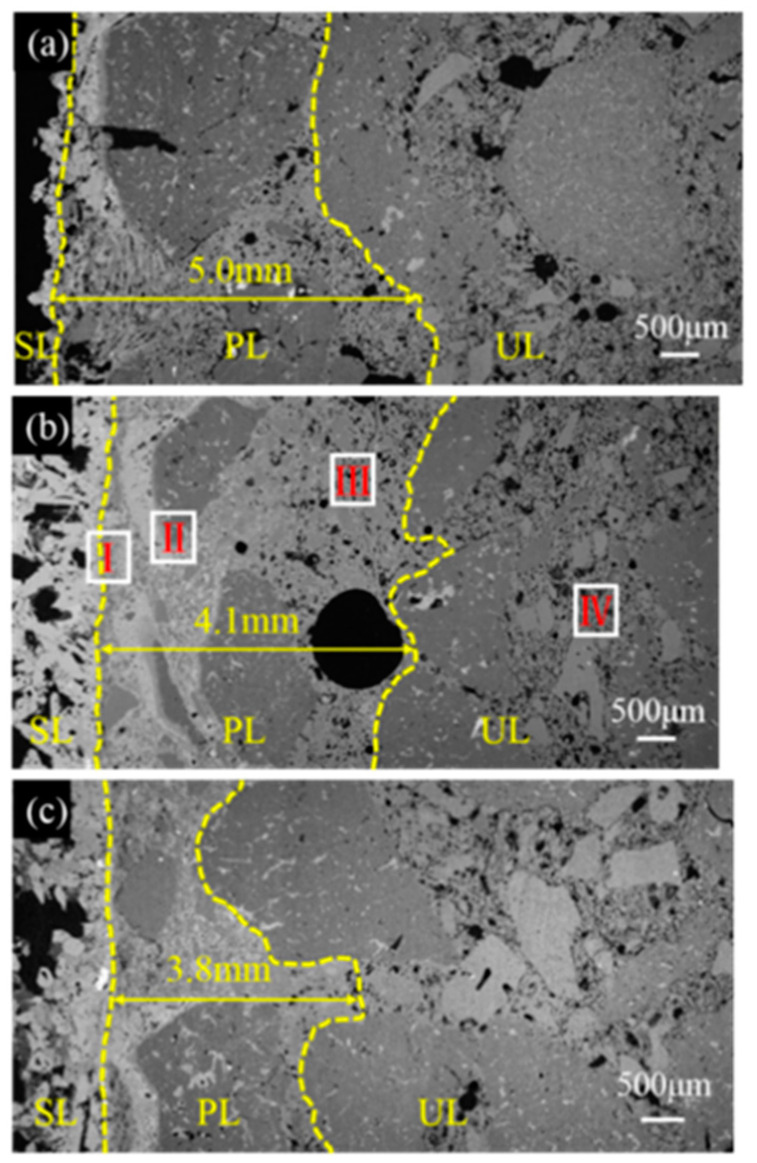
SEM images of sample (**a**) CB0.5, (**b**) CB1, and (**c**) CB2 after slag corrosion in reducing atmosphere.

The microstructural morphologies of the sample tested in the RA were determined by SEM and are shown in Figure 7. The penetrated depth is 5.0 mm, 4.1 mm, and 3.8 mm for sample CB0.5, CB1, and CB2, respectively, as presented in Figure 7, indicating that the slag resistance of the ASC castables tested in the RA is enhanced by raising the CB content.

**Figure 8:**
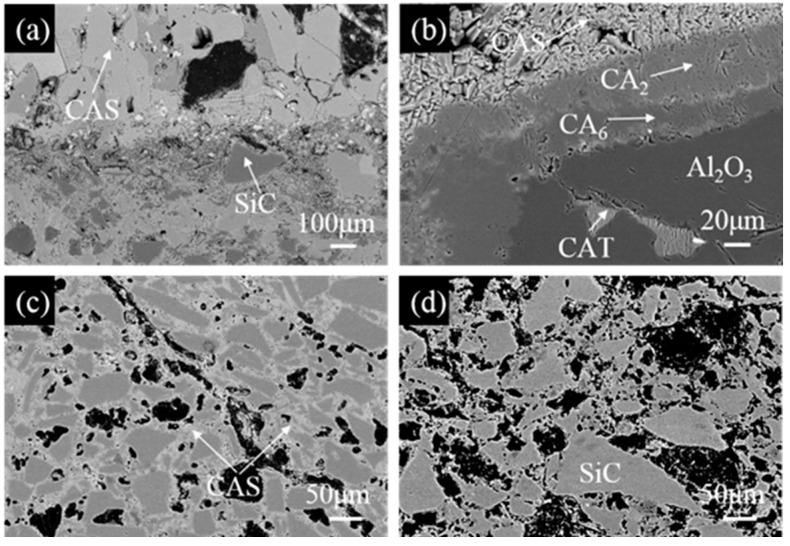
High-magnification images of (**a**) the slag/castable interface marked as square I, (**b**) the corrosion of fused alumina grain in the penetration layer marked as square II, (**c**) the penetration layer marked as square III, (**d**) the un-reacted layer marked as square IV in Figure 7b.

Figure 8 shows SEM images of the marked area (I–IV) in Figure 7b. From Figure 8a, it can be seen that the slag-to-refractory interface is clear, and a low melting point (anorthite, CAS) can be detected by EDS analysis in the penetrated layer, leading to the development of a dense structure. Except for SiC particles, other oxide substances are almost dissolved into the slag, indicating that SiC offers excellent fly ashes corrosion resistance. A continuous CA_2_ and CA_6_ dense layer is formed on the fused alumina grains (Figure 8b), creating a barrier against slags, which effectively prevents the slag from further infiltrating into alumina grains, thereby improving their fly ash resistance [23]. A low-melting-point phase (CaO⋅Al_2_O_3_⋅TiO_2_, CAT) was also found in brown alumina aggregates by slag penetration (gray), as shown in Figure 8b. As revealed in Figure 8c, quite a few CAS compounds were also detected. As for the un-reacted layer, shown in Figure 8d, quite a few pores (in black) are evenly distributed in the matrix. SiC grains are also observed, with sizes of about 50–100 μm.

### 3.3. Phase Composition and Microstructures of the Samples

**Figure 9:**
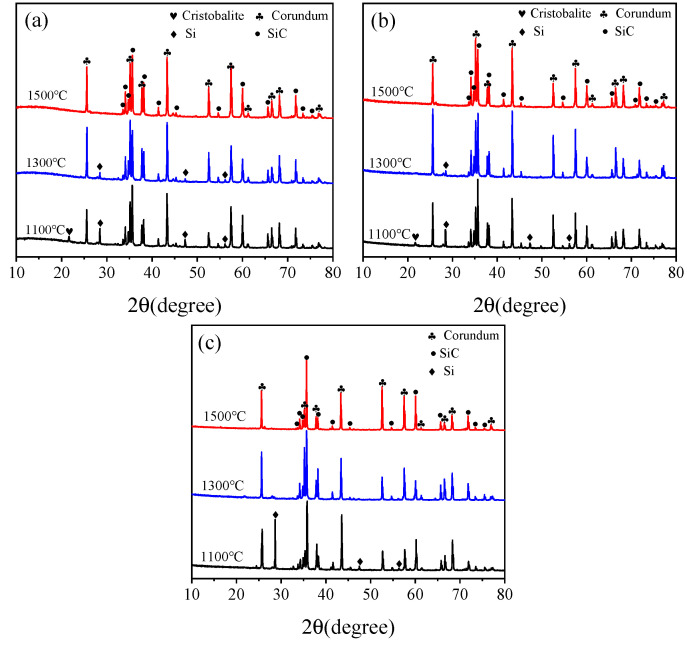
XRD patterns of sample CB1 after firing in the (**a**) OA, (**b**) WOA, and (**c**) RA.

Figure 9 depicts the XRD patterns of the sample CB1 matrix sintered in different treating atmospheres. As seen in Figure 9a, the phase composition of the specimen fired in the OA at 1100 °C includes corundum, SiC, Si, and tiny amount of cristobalite. At 1300 °C, Si peak heights decrease significantly and cristobalite disappears. At 1500 °C, Si disappears, and the phase composition comprises corundum and SiC. The phase composition of the matrix after firing in the WOA is not differed from that in the OA, shown in Figure 9b. The phase composition of the matrices pre-fired in the RA at 1100 °C comprises corundum, SiC, and Si, with no cristobalite detected; furthermore, the Si peak intensity is higher than that in the OA and WOA, as revealed in Figure 9c. After firing in the RA at 1300 °C and 1500 °C, the phase compositions are identical to those of castables fired in the OA and WOA. It can be noted that Si peaks completely disappeared at 1500 °C in the OA and WOA and at 1300 °C in the RA. This is ascribed to that CB is easily oxidized in the OA and WOA, and the oxygen partial pressure inside the sample is reduced, thereby protecting Si from being oxidized. CB is easily reacted with Si to form SiC primary crystals and whiskers; therefore, Si can be completely reacted with CB at 1300 °C in the RA.

**Figure 10:**
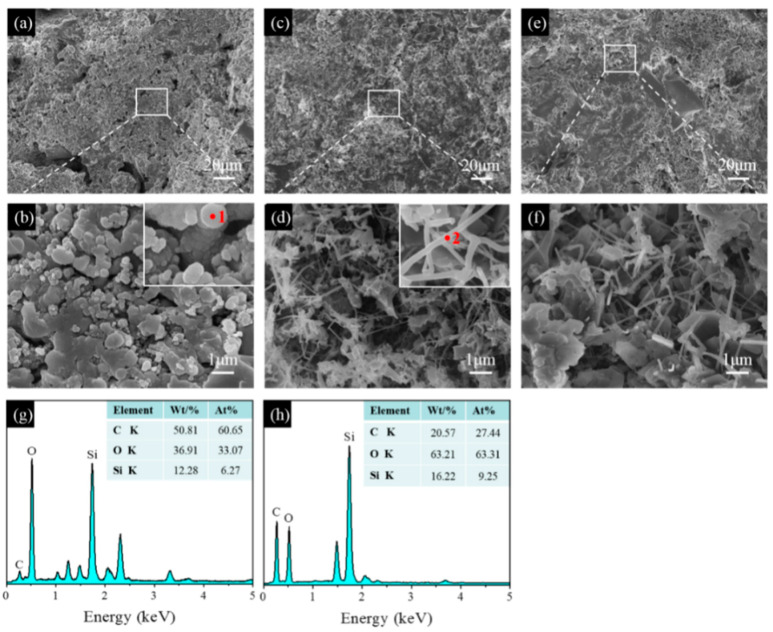
SEM images of sample CB1 fired in the OA at (**a**,**b**) 1100 °C, (**c**,**d**) 1300 °C, and (**e**,**f**) 1500 °C. (**g**) EDS spectrum of point 1, (**h**) EDS spectrum of point 2.

**Figure 11:**
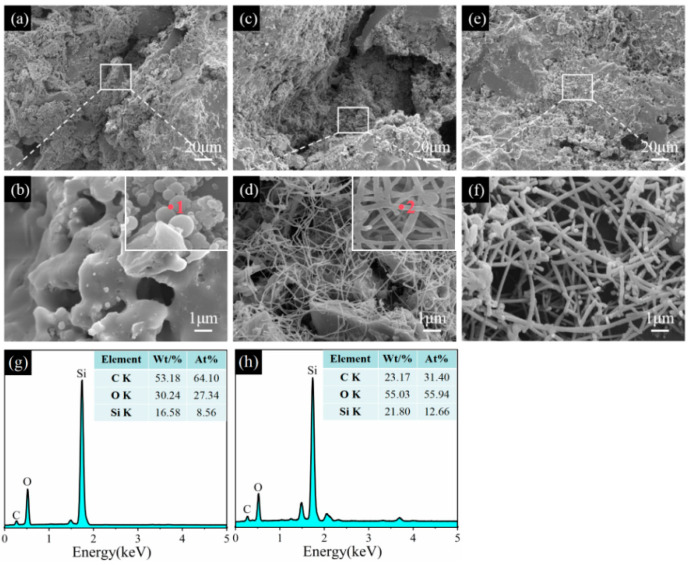
Morphologies of sample CB1 pre-fired at 1100 °C (**a**,**b**), 1300 °C (**c**,**d**), and 1500 °C (**e**,**f**) in the WOA. (**g**) EDS spectrum of point 1, (**h**) EDS spectrum of point 2.

**Figure 12:**
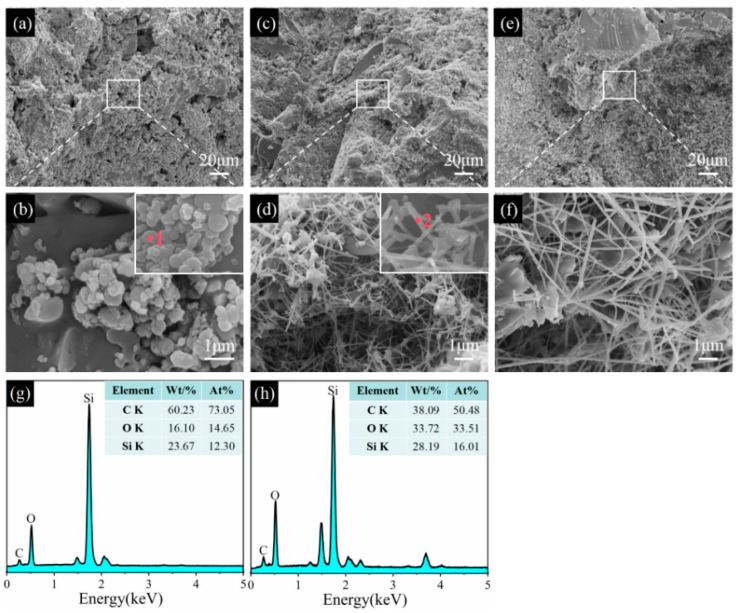
SEM images of sample CB1 fired in the RA at 1100 °C (**a**,**b**), 1300 °C (**c**,**d**), and 1500 °C (**e**,**f**). (**g**) EDS spectrum of point 1, (**h**) EDS spectrum of point 2.

SEM images of the samples pre-fired in the OA, WOA, and RA are shown in Figure 10, Figure 11 and Figure 12. After firing at 1100 °C in the OA, many agglomerated spheres with a diameter of 0.5–1 μm are evenly distributed in the matrix of sample CB1, shown in Figure 10a,b. The EDS results show that these aggregation spheres mainly consist of C, O, and Si elements, indicating that these spheres may be SiC primary crystals, which are formed in the reaction of CB with Si fines or gaseous SiO. At 1300 °C, many whisker-like compounds are found (Figure 10d), which are mainly composed of Si, C, and O elements and are identified by EDS analysis, indicating that these whiskers are SiC. By further increasing the temperature to 1500 °C, quantities of SiC whiskers are formed, and their aspect ratio is noticeably increased after firing at 1500 °C (Figure 10f).

Figure 11 shows SEM images of sample CB1 fired in the WOA. At 1100 °C in the OA, no agglomerated spheres are found in the matrix. At 1300 °C, many SiC whiskers with a diameter of about 50 nm are formed inside the sample (Figure 11c,d). EDS analysis shows that these aggregation spheres mainly consist of C, O, and Si elements (point 1, inset in Figure 11b), and the SiC whiskers are formed at 1300 °C, which has been confirmed by EDS analysis (point 2, inset in Figure 11d). By further increasing the temperature to 1500 °C, a number of SiC whiskers with a diameter of about 200 nm can be clearly observed, are interlaced with each other, and have developed a network structure (Figure 11f), leading to a strength improvement.

Regarding the sample CB1 sintered at 1100 °C in the RA, SiC primary grains are found and evenly distributed in the matrix, and their sizes are about 0.5 µm (Figure 12a,b). At 1300 °C and 1500 °C, SiC primary grains disappear, SiC whiskers (Figure 12d,f) are observed, and their aspect ratios are greatly increased as the temperature rises from 1300 °C to 1500 °C. EDS analysis shows that these aggregation spheres mainly consist of C, O, and Si elements (point 1, inset in Figure 12b, and these whiskers are composed of Si, O, and C elements (point 2, inset in Figure 12d), indicating that they are SiC. More importantly, SiC whiskers are mostly formed inside the pores and contribute to reducing their AP and enhancing their strength [23,28,33]. In comparison with the samples sintered in the OA and WOA, the amount and aspect ratios of SiC whiskers are noticeably increased and develop a continuous network, which is also beneficial to their TSR and slag resistance.

**Figure 13:**
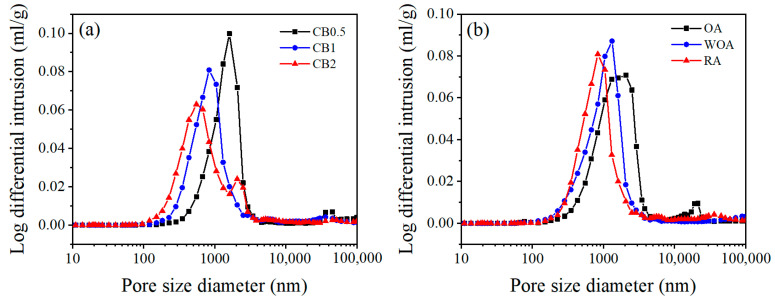
Pore size distribution of (**a**) the samples with different CB addition heat-treated in the RA and (**b**) sample CB1 fired in various sintering atmospheres at 1500 °C.

Figure 13 shows the pore characteristics of the samples pre-heated at 1500 °C. As illustrated in Figure 13a, pore sizes of the samples with different CB addition fired in the RA are mainly centered at 0.1–2 μm. Upon increasing the CB amount, the volume ratio of pore size (<1 μm) is significantly increased from 24.2% to 70.0%, the percentage of large pores (1–10 μm) is apparently reduced from 67.3% to 22.5%, and median pore diameter is reduced from 1.5 μm to 0.7 μm, as shown in Table 2. The volume ratio of the pore size (<1 μm) of sample CB1 pre-fired in the RA is the biggest (67.7%) and the median pore diameter (0.9 μm) is the smallest, and that in the OA is the smallest (28.0%) and the largest (1.5 μm), as illustrated in Table 3.

### 3.4. Discussion

The properties of the samples pre-heated in different sintering atmosphere are mainly influenced by their phase composition and microstructure, including the amount and morphologies of SiC whiskers and their pore size distribution [23,28], With CB rising from 0.5% to 1%, the CMOR of the specimens fired at 1500 °C in the RA is increased, which is ascribed to the merits of CB, a type of amorphous carbon with a large specific surface area, making it uniformly disperse in the matrix and fill into the pores in the castables, thereby densifying their structure and improving their strength. However, when CB is 2%, it adversely affects the continuity and sintering of the matrix because of the larger specific surface area, resulting in a further reduction in their cold strength, TSR (Figure 3), and hot strength (Figure 4). With the rising CB level, the corrosion resistance of the samples tested in the RA is noticeably enhanced, owing to more CB offering much better slag resistance, as well as to the smaller pore size and pore distribution in the samples (as seen in Figure 13 and Table 2).

The CMOR of the samples fired in the OA and RA at 1500 °C is higher (about 20 MPa), and that in the WOA is the lowest (about 16.8 MPa). The TSR of the sample fired in the RA is the best (RSR: 85.4%), and the smallest value (RSR: 56.7%) is found for sample CB1 when pre-fired in the OA. The HMOR of the sample tested in the OA is the highest (5.3 MPa), and the HMOR measured in the WOA is the lowest (1.5 MPa). The corrosion resistance of the sample tested in the OA is the best (the penetration depth was 2.5 mm) due to the liquid SiO_2_ formed by the oxidation of SiC grains, which can react with Al_2_O_3_ to form mullite, thereby creating a strengthening effect [23,28,33]. However, this is detrimental to the TSR.

Compared to the samples fired in the OA, WOA, and RA, SiC whiskers formed in the RA have a higher aspect ratio and quantity, and they finally develop a continuous network structure, which may be due to CB particles having a smaller particle size, higher activity, and larger specific surface area, thereby enhancing their cold strength, TSR, and slag resistance. However, Si and CB are easily oxidized in the OA and WOA, and the formation of SiC is adversely affected; therefore, the number of SiC whiskers is lower than that in the RA. Furthermore, quantities of SiC primary crystals are formed in the sample pre-fired in the RA (Figure 12b) and are also conducive to the formation of SiC whiskers at high temperatures (Figure 12f); in addition, their pore sizes are also reduced, which is beneficial in enhancing the TSR and slag resistance of the castables [28,33].

## 4. Conclusions

ASC castables were fabricated using CB as carbon source, and the influences of the CB amount and firing atmosphere on the properties of ASC castables were investigated. The results were as follows:

(1) On increasing the CB content from 0.5% to 2%, the cold strength and slag resistance of the ASC castables heat-treated in the RA are slightly increased; however, their porosity, hot strength, and thermal shock resistance are slightly reduced. After sintering in the OA, the cold strength, slag resistance and thermal shock resistance of the samples are slightly increased, but their hot strength is reduced. As for the samples fired in the WOA, the cold strength and hot strength are reduced; however, their thermal shock resistance and slag resistance gradually increase.
(2) Regarding the samples with 1% CB addition, the CMOR values of the samples fired in the OA and RA at 1500 °C are higher (about 20 MPa), the TSR of the samples fired in the RA is the best (RSR: 85.4%), the HMOR of the samples tested in the OA is the highest (5.3 MPa), and the slag resistance of the samples in the OA is the best. The merit of the castables with 1% CB is in the RA > in the OA > in the WOA.
(3) This is ascribed to the fact that more SiC whiskers form in the RA with a larger aspect ratio and finally develop a continuous network structure, thereby enhancing their cold strength, TSR, and slag resistance. The cold strength, hot strength, and slag resistance of sample CB1 tested in the OA are the highest, due to the liquid SiO_2_ formed by the oxidation of SiC being able to fill the pores and react with Al_2_O_3_ to form mullite, creating a strengthening effect; however, these effects are detrimental to the TSR.

## Figures and Tables

**Table 1:** Formulations of ASC castables (wt%).

Raw Materials	Sample No.			Supplier
	CB0.5	CB1	CB2	
Brown alumina	55	55	55	Taiyue Abrasive Co., Qinyuan, China
SiC	33	32.5	31.5	Yuancheng Co., Qinyang, China
Micro-powders	8	8	8	Kaifeng Tenai Co., Kaifeng, China, Elkem Co., Shanghai, China
Si fines	2	2	2	Rixin Co., Guangzhou, China
Cement	1.5	1.5	1.5	Imery Co., Tianjin, China
CB	0.5	1	2	Cofermin Co., Tianjin, China
FS 20	+0.2	+0.2	+0.2	BASF Co., Ludwigshafen, Germany

**Table 2:** Pore characteristics of the samples pre-fired in the RA at 1500 °C.

Samples		CB0.5	CB1	CB2
Pore size distribution/%	<1 μm	24.2	67.7	70
	1–10 μm	67.3	24.9	22.5
	>10 μm	8.5	7.4	7.5
Median pore diameter/μm		1.5	0.9	0.7

**Table 3:** Pore characteristics of sample CB1 fired in various atmospheres.

Firing Atmosphere		OA	WOA	RA
Pore size distribution/%	<1 μm	28.0	46.6	67.7
	1–10 μm	65.0	46	24.9
	>10 μm	7.0	7.4	7.4
Median pore diameter/μm		1.5	1.1	0.9

## Data Availability

Date are contained within the article.

## Acronyms

CB	Carbon black
RA	Reducing atmosphere
OA	Oxidizing atmosphere
WOA	Weak-oxidizing atmosphere
BD	Bulk density
AP	Apparent porosity
ASC	Al_2_O_3_-SiC-C
RSR	Residual strength ratio
PLC	Permanent linear change
TSR	Thermal shock resistance
HMOR	Hot modulus of rupture
CMOR	Cold modulus of rupture
CMORst	Residual strength
SL	Slag layer
PL	Slag penetrated layer
UL	Uncorroded layer

## References

[1] Ministry of Ecology and Environment of The People’s Republic of China (2020). Annual Report on Solid Waste Pollution Prevention and Control in Large and Medium Sized Cities in China.

[2] National Bureau of Statistics of China (2019). China Statistical Yearbook of 2019.

[3] Prompt N., Ouedraogo E. (2008). High temperature mechanical characterisation of an alumina refractory concrete for Blast Furnace main trough: Part I. General context. J. Eur. Ceram. Soc..

[4] Wang W., Jia M., Che X. (2020). Pretreatment of municipal solid waste incineration fly ash and preparation of solid waste source sulphoaluminate cementitious material. J. Hazard. Mater..

[5] Li X., Zhang C., Li Y., Zhi Q. (2016). The status of municipal solid waste incineration (MSWI) in China and its clean development. Energy Procedia..

[6] Sun X., Li J., Zhao X., Zhu B., Zhang G. (2016). A review on the management of municipal solid waste fly ash in American. Procedia Environ. Sci..

[7] Zacco A., Borgese L., Gianoncelli A., Struis R.P., Depero L.E., Bontempi E. (2014). Review of fly ash inertisation treatments and recycling. Environ. Chem. Lett..

[8] Lindberg D., Molin C., Hupa M. (2015). Thermal treatment of solid residues from WtE units: A review. Waste Manag..

[9] Gu Q., Wu W., Jin B. (2019). Investigation of thermal characteristics of municipal solid waste incineration fly ash under various atmospheres: A TG-FTIR study. Thermochim. Acta..

[10] Zhao P., Ni G., Jiang Y., Chen L., Chen M., Meng Y. (2010). Destruction of inorganic municipal solid waste incinerator fly ash in a DC arc plasma furnace. J. Hazard. Mater..

[11] Yang G.C., Chuang T.N., Huang C.W. (2017). Achieving zero waste of municipal incinerator fly ash by melting in electric arc furnaces while steelmaking. Waste Manag..

[12] Pan X., Yan J., Xie Z. (2013). Detoxifying PCDD/Fs and heavy metals in fly ash from medical waste incinerators with a DC double arc plasma torch. J. Environ. Sci..

[13] Sperber J., Burgard R., Duennes F.J. (2012). Innovative lining concepts for hazardous waste incineration. Refract. World Forum..

[14] Chen D., Huang A., Gu H., Zhang S., Shao Z. (2015). Corrosion of Al_2_O_3_–Cr_2_O_3_ refractory lining for high-temperature solid waste incinerator. Ceram. Int..

[15] Chen D., Gu H., Huang A. (2019). Towards chrome-free lining for plasma gasifiers using the CA_6_-SiC castable based on high-temperature water vapor corrosion. Ceram. Int..

[16] Chen D., Gu H., Huang A., Shao Z. (2017). Towards chrome-free of high-temperature solid waste gasifier through in-situ SiC whisker enhanced silica sol bonded SiC castable. Ceram. Int..

[17] Bie C., Sang S., Li Y., Zhu T., Xu Y. (2016). Research of Al_2_O_3_-SiC-C refractories as chromia-free lining for gasifier. Ceram. Int..

[18] Liu Z., Deng C., Yu C., Wang X., Ding J., Zhu H. (2019). Molten salt synthesis and characterization of SiC whiskers containing coating on graphite for application in Al_2_O_3_-SiC-C castables. J. Alloys Compd..

[19] Zhao L., Yin Y., Yao N., Ma H., Zhang S., Liu G., Jia Q. (2023). Preparation of core-shell SiC@ C powder via modified molten salt shielding technique. J. Chin. Ceram. Soc..

[20] Yin Y., Wang S., Zhang S., Cui J., Liu X., Jia Q. (2022). Preparation of SiC coated graphite flake with much improved performance via a molten salt shielded method. Int. J. Appl. Ceram. Tec..

[21] Li Y., Yin Y., Chen J., Kang S., Ma H., Zhang S., Jia Q. (2023). Large-Scale Fabrication of SiC-TiC@ C Powders via Modified Molten Salt Shielding Synthesis Technique and Their Effect on the Properties of Al_2_O_3_-MgO Castables. Materials.

[22] Zhao L., Yin Y., Li Y., Ma H., Liu X., Zhang S., Jia Q. (2023). Large-scale fabrication of TiC@ C powders and its effect on the properties of Al_2_O_3_-MgO-C castables. Int. J. Appl. Ceram. Tec..

[23] Wang S., Zhou P., Liu X., Cui J., Liu X., Zhang S., Jia Q. (2022). Effect of modified coal tar pitch addition on the microstructure and properties of Al_2_O_3_-SiC-C castables for solid waste incinerators. Ceram. Int..

[24] Li S., Liu J., Wang J., Han L., Zhang H., Zhang S. (2018). Catalytic preparation of graphitic carbon spheres for Al_2_O_3_-SiC-C castables. Ceram. Int..

[25] Zang Y., Xiao G., Ding D., Chen J., Lei C., Luo J., Chong X. (2022). Study on cobweb-like carbon nanotubes/calcium aluminate cement and its effect on the properties of Al_2_O_3_–SiC–C castables. Int. J. Appl. Ceram. Tec..

[26] Wang J., Li Q., Wu C., Xu H. (2014). Thermal conductivity and mechanical properties of carbon black filled silicone rubber. Polym Compos..

[27] Wu M., Huang A., Yang S., Gu H., Fu L., Li G., Dong H. (2020). Corrosion mechanism of Al_2_O_3_–SiC–C refractory by SiO_2_-MgO-based slag. Ceram. Int..

[28] Zhou P., Qiu X., Luo Z., Liu X., Zhang S., Jia Q. (2021). Effect of firing atmosphere on the microstructure and properties of Al_2_O_3_-SiC-C castables. Ceram. Int..

[29] Li X., Li Y., Chen L., Zhu B. (2015). Matrix structure evolution and thermomechanical properties of carbon fiber-reinforced Al_2_O_3_-SiC-C castable composites. Mater. Res. Bull..

[30] Long T., Gu H., Zhang M., Huang A., Jiang Q., Fu L., Chen D., Cao J., Li L., Qiu W. (2024). Effect of pretreated Al-Si alloy powder on the microstructure and properties of Al_2_O_3_-SiC-C castables for iron runner. J. Alloys Compd..

[31] Li Y., Zhao H., Zhang H., Pan D., Feng Y., Li Y., Wang X., Guo Y. (2019). Enhancement and explosion-proof mechanism of aluminum fiber addition in Al_2_O_3_-SiC-C castables for iron runner. Ceram. Int..

[32] Yang Y., Liu H., Wang Z., Ma Y., Wang X. (2023). Microstructure and enhanced slag resistance of Al_2_O_3_–SiC–C refractory castables with addition of ammonium metatungstate. Ceram. Int..

[33] Lian J., Zhu B., Li X., Chen P., Fang B. (2016). Effect of in situ synthesized SiC whiskers and mullite phases on the thermo-mechanical properties of Al_2_O_3_-SiC-C refractories. Ceram. Int..

